# Posterior Cervicothoracic Fusion During Dorsal Root Entry Zone Lesioning for Brachial Plexus Avulsion: Sagittal Alignment and Deformity Outcomes

**DOI:** 10.1227/neuprac.0000000000000278

**Published:** 2026-08-07

**Authors:** Stephen M. Bergin, Joshua Woo, Mohamed Alwadai, Benjamin S. Succop, Blake Parente, Peter G. Passias, Allan H. Friedman, Shivanand P. Lad, Muhammad M. Abd-El-Barr

**Affiliations:** 1 Department of Neurosurgery, Duke University Medical Center, Durham, North Carolina, USA;; 2 Department of Orthopaedic Surgery, Duke University Medical Center, Durham, North Carolina, USA

**Keywords:** Brachial plexus avulsion, Cervical fusion, Dorsal root entry zone, Kyphosis, Neuropathic pain, Sagittal alignment, Spine deformity

## Abstract

**BACKGROUND AND OBJECTIVES:**

Dorsal root entry zone (DREZ) lesioning is an established treatment of refractory deafferentation pain after brachial plexus avulsion. Exposure often requires multilevel cervical or cervicothoracic laminectomy, which may predispose to postoperative kyphosis. We evaluated whether concurrent posterior instrumented fusion preserves sagittal alignment and reduces delayed deformity after DREZ lesioning.

**METHODS:**

We retrospectively reviewed patients undergoing cervical or cervicothoracic DREZ lesioning at a single institution from 2013 to 2025. Patients were grouped by concurrent posterior fusion vs nonfusion DREZ. Radiographic outcomes included C2-C7 Cobb angle, cervical sagittal vertical axis (CSVA), T1 slope, C2 slope, and T1 slope minus cervical lordosis. Nonfusion patients with paired standing radiographs underwent exploratory comparison. Clinical outcomes included pain relief, complications, and reoperation.

**RESULTS:**

Twenty-three patients were included: 8 fusion and 15 nonfusion. In the fusion cohort, median follow-up was 16.0 (IQR: 10.9-21.4). Paired standing radiographs were available in 7 of 8 fusion patients and 3 of 15 nonfusion patients. In the fusion cohort, mean C2-C7 Cobb improved from 2.5° ± 23.1° to 18.3° ± 20.8°, whereas CSVA remained stable. All fusion patients maintained postoperative CSVA ≤40 mm, and 7 of 8 maintained T1 slope minus cervical lordosis ≤20°. No fusion patient developed new or progressive postoperative kyphosis. Durable pain relief occurred in 5 of 8 fusion patients and 10 of 15 nonfusion patients. In the nonfusion cohort, postoperative kyphosis was documented in 8 of 15 patients, new or progressive kyphosis/deformity in 5 of 12 evaluable patients, and reoperation for deformity, instability, or radiographic failure in 5 of 15.

**CONCLUSION:**

Concurrent posterior fusion during cervicothoracic DREZ lesioning was associated with preserved sagittal alignment and absence of new progressive postoperative kyphosis. Fusion should be considered when DREZ requires extensive multilevel exposure, particularly across the cervicothoracic junction.

**ABBREVIATION:**
CSVA: cervical sagittal vertical axis
DREZ: dorsal root entry zone
TS-CL: T1 slope minus cervical lordosis.

Dorsal root entry zone (DREZ) lesioning is an established microsurgical treatment of medically refractory neuropathic pain, particularly after brachial plexus avulsion or *other* central deafferentation injuries.^1,2^ The procedure targets hyperactive neurons and pain-generating circuitry within the dorsal horn at the DREZ.^3,4^ Adequate exposure often requires multilevel posterior cervical or cervicothoracic decompression, historically performed through laminectomy or hemilaminectomy.^5^ Although hemilaminectomy may reduce disruption of the posterior tension band and is well suited for unilateral pathology, full laminectomy provides broader exposure of the dorsal cord and may facilitate DREZ identification in patients with distorted traumatic anatomy.^6,7^

The structural consequences of posterior exposure are especially relevant in patients with chronic brachial plexus avulsion. These injuries frequently result from high-energy traction mechanisms and may be associated with long-standing denervation, shoulder girdle imbalance, paraspinal muscular atrophy, and asymmetric cervicothoracic loading. As a result, cervical malalignment may develop during the chronic postinjury interval, before DREZ lesioning is performed. Multilevel posterior decompression across the cervicothoracic junction may further compromise posterior stabilizing elements and increase the risk of progressive kyphosis, instability, or delayed deformity correction.^8,9^

At our institution, DREZ lesioning was historically performed without concurrent instrumentation.^10,11^ After observing delayed cervical kyphosis, instability, and deformity-related revision surgery in patients treated without fusion (Figure 1), our operative strategy evolved to include posterior instrumented fusion when DREZ exposure required multilevel decompression across the cervicothoracic junction or when preoperative alignment suggested structural vulnerability. The rationale was not solely prophylaxis against postlaminectomy kyphosis, but stabilization of a spine already at risk because of chronic deafferentation injury, muscular imbalance, and deformity progression.

**FIGURE 1.**
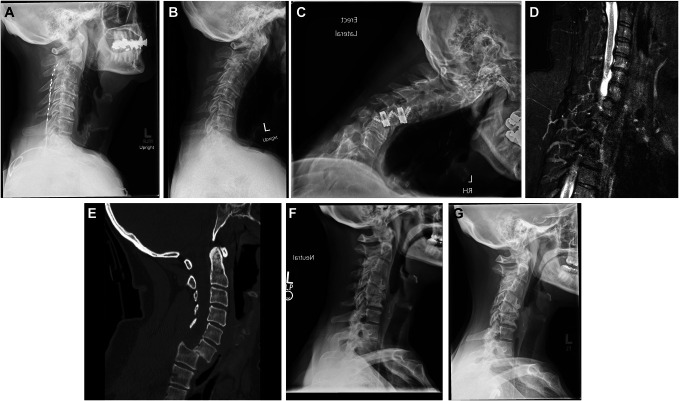
Representative radiographic failures after DREZ lesioning without concurrent fusion. **A**-**C**, Progressive postlaminectomy kyphotic deformity. A 72-year-old man with right-hand phantom pain after traumatic amputation and brachial plexus avulsion 24 years before. **A**, Preoperative lateral radiograph demonstrating loss of cervical lordosis with previous spinal cord stimulator leads. C2-C7 Cobb angle −4°, CSVA 25.4 mm, T1 slope 5°, C2 slope 4°. **B**, Lateral radiograph 8 months after C4-C7 hemilaminectomy and DREZ lesioning demonstrating progressive kyphosis. C2-C7 Cobb angle −35°, CSVA 48.7 mm, T1 slope 7°, C2 slope 47°. **C**, Lateral radiograph 2 years after index DREZ lesioning demonstrating severe sagittal malalignment despite interval outside-hospital anterior cervical discectomy and fusion. C2-C7 Cobb angle 44°, CSVA 99.2 mm, T1 slope 30°, C2 slope 78°. **D** and **E**, Delayed segmental instability. A 59-year-old woman with left brachial plexus avulsion after motor vehicle collision 4 years before. **D**, Preoperative short-TI inversion recovery MRI obtained using brachial plexus protocol. **E**, Computed tomography cervical spine obtained after presentation to the emergency department 5 months after C5-T1 hemilaminectomy and DREZ lesioning, demonstrating delayed postoperative segmental instability. **F** and **G**, Early kyphosis progression. A 43-year-old man with left brachial plexus avulsion 15 years before. **F**, Preoperative lateral cervical radiograph. C2-C7 Cobb angle −7°, CSVA 26.6 mm, T1 slope 8°, C2 slope 17°. **G**, Lateral radiograph 5 months after C3-C6 laminectomies for DREZ lesioning demonstrating early kyphotic progression. C2-C7 Cobb angle −12°, CSVA 32.6 mm, T1 slope 9°, C2 slope 19°. CSVA, cervical sagittal vertical axis; DREZ, dorsal root entry zone.

The role of concurrent fusion during DREZ lesioning remains poorly defined. Fusion may preserve sagittal alignment and prevent delayed structural failure, but it also increases operative magnitude, reduces segmental motion, and introduces risks specific to instrumentation.^12^ Whether these tradeoffs are justified in patients undergoing DREZ lesioning for severe refractory deafferentation pain has not been well studied.

In this study, we retrospectively compared patients who underwent DREZ lesioning with concurrent posterior cervicothoracic fusion to a historical cohort treated with DREZ lesioning without fusion. We evaluated postoperative sagittal alignment, delayed kyphosis or instability, deformity-related reoperation, pain outcomes, and complications. We further described representative cases demonstrating progressive postinjury kyphosis before DREZ lesioning and delayed deformity after nonfusion DREZ, illustrating the structural rationale for selective concurrent stabilization.

## METHODS

This retrospective cohort study was approved by the institutional review board with waiver of informed consent. Patients were stratified by operative strategy into those treated with concurrent posterior instrumented fusion and those treated without fusion.

### Patient Selection

Patients were included if they underwent cervical or cervicothoracic DREZ lesioning for medically refractory neuropathic pain secondary to brachial plexus avulsion, traumatic deafferentation pain, or central deafferentation syndrome. Patients were excluded if operative records or clinical follow-up were unavailable. The fusion cohort included patients who underwent DREZ lesioning with concurrent posterior cervical or cervicothoracic instrumented fusion. The nonfusion cohort included patients who underwent DREZ lesioning without concurrent fusion, including hemilaminectomy, full laminectomy, or redo posterior decompression exposures.

### Surgical Technique

Patients were positioned prone in a Mayfield head holder with continuous neurophysiological monitoring. In fusion cases, posterior cervical or cervicothoracic instrumentation was placed using cervical lateral mass and thoracic pedicle screws, followed by multilevel laminectomy for dorsal cord exposure. Under microscopic visualization, the dura was opened and the DREZ was localized along the dorsolateral sulcus using anatomic landmarks, neuromonitoring, and test stimulation. Radiofrequency lesions were created with a Diros electrode at 75°C for 15 seconds per lesion, placed at 1- to 2-mm intervals, and directed ventromedially into the dorsal horn toward the substantia gelatinosa. Motor-evoked potentials were assessed throughout lesioning. After watertight dural closure, posterior arthrodesis was performed using rods, local autograft, and demineralized bone matrix.

### Data Collection

Demographic, injury, operative, radiographic, clinical outcome, complication, and reoperation variables were abstracted from the medical record. Baseline variables included age, sex, body mass index, smoking status, American Society of Anesthesiologists class, medical comorbidities, injury mechanism, laterality, avulsion status, preoperative limb function, and time from injury to DREZ. Operative variables included exposure type, levels exposed, cervicothoracic junction involvement, DREZ levels, number of lesions, fusion levels, and concurrent procedures.

### Outcomes

Clinical outcomes included durability of pain relief (durable, partial, none, or recurrence), changes in analgesic requirements, neurological function, and need for reoperation. Fusion status was assessed radiographically at last follow-up. In the nonfusion cohort, deformity outcomes were assessed based on documented clinical instability, radiographic reports, and subsequent need for delayed fusion or revision surgery.

### Radiographic Measurements

Standing lateral cervical radiographs were reviewed preoperatively and at final radiographic follow-up when available. Cervical sagittal alignment was assessed using previously described parameters, including C2-C7 Cobb angle, cervical sagittal vertical axis (CSVA), T1 slope, C2 slope, and T1 slope minus cervical lordosis (TS-CL).^13,14^ C2-C7 Cobb angle was recorded using a lordosis-positive convention; negative values indicated kyphosis. TS-CL was calculated as T1 slope minus C2-C7 lordosis, and change was calculated as postoperative minus preoperative value.

In the nonfusion cohort, quantitative analysis was limited by heterogeneous imaging availability. When standing radiographs were unavailable, measurements were obtained from MRI, computed tomography, or cross-table radiographs when technically feasible. Deformity events were also identified from radiology reports, outside imaging documentation, clinical notes, and delayed deformity or instability surgery.

### Statistical Analysis

Continuous variables are reported as mean ± SD or median (IQR), as appropriate. Categorical variables are reported as n/N (%). Between-cohort comparisons used Fisher exact tests for categorical variables and Mann-Whitney *U* tests for continuous variables. *P* values for continuous radiographic variables compared change scores between the fusion cohort and nonfusion paired X-ray subset. Statistical analyses were considered exploratory.

## RESULTS

A total of 23 patients underwent cervical or cervicothoracic DREZ lesioning between 2013 and 2025 and met inclusion criteria. Eight patients underwent DREZ lesioning with concurrent posterior instrumented fusion, and 15 underwent DREZ lesioning without fusion. Baseline demographic and injury characteristics are summarized in Table 1. The fusion cohort was younger than the nonfusion cohort and consisted entirely of patients with complete traumatic brachial plexus avulsion. Motorcycle collision was the most common mechanism in the fusion cohort.

**TABLE 1.** Baseline Demographics and Injury Characteristics

Variable	Fusion cohort, n = 8	Nonfusion cohort, n = 15	Difference, fusion minus nonfusion (95% CI)	*P* value
Age at surgery, y, mean ± SD	42.1 ± 11.3	56.9 ± 11.6	Mean difference −14.8 y (−25.5 to −4.1)	**.018**
Male sex, n/N (%)	8/8 (100)	12/15 (80.0)	Risk difference +20.0% (−14.9 to 45.2)	.526
BMI, mean ± SD	29.8 ± 6.3	29.4 ± 6.8	Mean difference +0.4 kg/m^2^ (−5.6 to 6.4)	.825
ASA class ≥3, n/N (%)	3/8 (37.5)	12/15 (80.0)	Risk difference −42.5% (−69.6 to −1.8)	.071
Ever smoker, n/N (%)	4/8 (50.0)	6/15 (40.0)	Risk difference +10.0% (−27.4 to 44.9)	.685
Diabetes, n/N (%)	1/8 (12.5)	7/15 (46.7)	Risk difference −34.2% (−59.5 to 6.7)	.176
Osteoporosis, n/N (%)	0/8 (0)	0/15 (0)	Risk difference 0.0% (−20.4 to 32.4)	>.999
Chronic steroid use, n/N (%)	0/8 (0)	1/15 (6.7)	Risk difference −6.7% (−29.8 to 26.2)	>.999
Left-sided injury, n/N (%)	4/8 (50.0)	10/15 (66.7)	Risk difference −16.7% (−50.4 to 21.2)	.657
Motorcycle collision, n/N (%)	6/8 (75.0)	3/15 (20.0)	Risk difference +55.0% (12.6 to 77.1)	**.023**
Vehicle-related trauma, n/N (%)	8/8 (100)	9/15 (60.0)	Risk difference +40.0% (1.8 to 64.3)	.058
Brachial plexus avulsion, n/N (%)	8/8 (100)	12/15 (80.0)	Risk difference +20.0% (−14.9 to 45.2)	.526
Complete avulsion, n/N (%)	8/8 (100)	12/15 (80.0)	Risk difference +20.0% (−14.9 to 45.2)	.526
Time from injury to DREZ, mo, median (IQR)	80.3 (36.9-168.9)	128.0 (63.4-288.1)	Median difference −47.8 mo (−224.8 to 105.6)	.220
Preoperative limb status				
Flail or amputated extremity, n/N (%)	8/8 (100)	10/15 (66.7)	Risk difference +33.3% (−3.8 to 58.3)	.122
Partial residual function after reconstruction, n/N (%)	0/8 (0)	3/15 (20.0)	Risk difference −20.0% (−45.2 to 14.9)	.526
Partial function without reconstruction/other, n/N (%)	0/8 (0)	2/15 (13.3)	Risk difference −13.3% (−37.9 to 20.5)	.526

ASA, American Society of Anesthesiologists; BMI, body mass index; DREZ, dorsal root entry zone.

Continuous variables are reported as mean ± SD unless otherwise specified. Time from injury to DREZ is reported as median (IQR). Categorical variables are reported as n/N (%). Effect estimates are reported as mean differences for continuous variables summarized as mean ± SD, risk differences for categorical variables, and median difference for time from injury to DREZ. Risk differences are reported as fusion minus nonfusion using Newcombe 95% CIs. Mean differences use Welch 95% CIs. The median difference for time from injury to DREZ is reported with a bootstrap 95% CI. *P* values are exploratory and were not adjusted for multiple comparisons. Ever smoking includes current and previous smokers. Vehicle-related trauma included motorcycle, motor vehicle, bicycle, pedestrian-versus-motor-vehicle, and snowmobile injuries. Complete avulsion excludes partial avulsion and nonavulsion etiologies. Partial residual function includes documented function after previous nerve transfer, muscle transfer, or reconstruction. Flail/amputated extremity includes patients documented as having a flail extremity or previous amputation. Bold denotes *P* value less than .05, indicating statistical significance.

### Operative Characteristics and Complications

Operative characteristics and complications are summarized in Table 2. The fusion cohort underwent more extensive posterior exposure, with full laminectomy and bilateral decompression performed in all cases. All fusion constructs crossed the cervicothoracic junction. By contrast, the nonfusion cohort included a mixture of hemilaminectomy and full laminectomy exposures, with fewer bilateral exposures and fewer cases crossing the cervicothoracic junction.

**TABLE 2.** Operative Characteristics and Complications

Variable	Fusion cohort, n = 8	Nonfusion cohort, n = 15	Difference, fusion minus nonfusion (95% CI)	*P* value
Full laminectomy exposure, n/N (%)	8/8 (100)	7/15 (46.7)	Risk difference +53.3% (13.4 to 75.2)	**.019**
Bilateral exposure, n/N (%)	8/8 (100)	5/15 (33.3)	Risk difference +66.7% (25.7 to 84.8)	**.003**
No. of levels exposed, mean ± SD	5.2 ± 1.3	4.0 ± 1.2	Mean difference +1.2 levels (0.01 to 2.4)	**.034**
Crossed cervicothoracic junction, n/N (%)	8/8 (100)	10/15 (66.7)	Risk difference +33.3% (−3.8 to 58.3)	.122
No. of DREZ lesions, mean ± SD	20.8 ± 3.4	28.1 ± 13.5	Mean difference −7.3 lesions (−15.2 to 0.6)	.458
CSF leak, n/N (%)	1/8 (12.5)	2/15 (13.3)	Risk difference −0.8% (−27.4 to 35.1)	>.999
Pseudomeningocele/CSF collection, n/N (%)	1/8 (12.5)	2/15 (13.3)	Risk difference −0.8% (−27.4 to 35.1)	>.999
Instrumentation failure, n/N (%)	1/8 (12.5)	0/15 (0)	Risk difference +12.5% (−10.3 to 47.1)	.348
New neurological deficit, n/N (%)	0/8 (0)	2/15 (13.3)	Risk difference −13.3% (−37.9 to 20.5)	.526
Wound complication, n/N (%)	0/8 (0)	3/15 (20.0)	Risk difference −20.0% (−45.2 to 14.9)	.526
Reoperation required, n/N (%)	2/8 (25.0)	5/15 (33.3)	Risk difference −8.3% (−39.0 to 30.3)	>.999

CSF, cerebrospinal fluid; DREZ, dorsal root entry zone.

Categorical variables are reported as n/N (%) and continuous variables as mean ± SD. Effect estimates are reported as risk differences for categorical variables and mean differences for continuous variables, calculated as fusion minus nonfusion. Risk differences use Newcombe 95% CIs. Mean differences use Welch 95% CIs. *P* values are exploratory and were not adjusted for multiple comparisons. Number of DREZ lesions was available for 6 fusion patients and 15 nonfusion patients. Instrumentation failure refers to pedicle screw fracture. CSF leak and pseudomeningocele/CSF collection were reported separately from wound complications unless wound drainage, dehiscence, infection, or washout was documented. Reoperation included surgery for CSF diversion/collection, instrumentation failure, instability, deformity, or pain salvage. Bold denotes *P* value less than .05, indicating statistical significance.

Complication rates were similar between cohorts. One fusion patient developed a cerebrospinal fluid (CSF) leak/pseudomeningocele requiring CSF diversion, and one developed instrumentation failure from pedicle screw fracture requiring revision. In the nonfusion cohort, CSF leak/pseudomeningocele, wound complications, and new neurological deficits were observed, as summarized in Table 2. Overall reoperation rates were 25.0% in the fusion cohort and 33.3% in the nonfusion cohort.

### Pain Outcomes

Pain outcomes are summarized in Table 3. Durable pain relief occurred in 5 of 8 fusion patients (62.5%) and 10 of 15 nonfusion patients (66.7%). Complete durable relief was more common in the fusion cohort, whereas partial durable relief was more common in the nonfusion cohort. Transient relief/recurrence and no meaningful improvement occurred at similar rates. Medication reduction was documented in 5 fusion patients (62.5%) and 11 nonfusion patients (73.3%). Overall, pain outcomes were similar between cohorts.

**TABLE 3.** Pain Outcomes

Variable	Fusion cohort, n = 8	Nonfusion cohort, n = 15	Difference, fusion minus nonfusion (95% CI)	*P* value
Clinical follow-up, mo, median (IQR)	16.0 (10.9-21.4)	11.6 (3.4-23.5)	Median difference +4.4 mo (−10.2 to 17.7)	.428
Any durable pain relief, n/N (%)	5/8 (62.5)	10/15 (66.7)	Risk difference −4.2% (−40.9 to 30.3)	>.999
Complete durable pain relief, n/N (%)	4/8 (50.0)	3/15 (20.0)	Risk difference +30.0% (−8.0 to 61.3)	.182
Partial durable pain relief, n/N (%)	1/8 (12.5)	5/15 (33.3)	Risk difference −20.8% (−47.8 to 18.2)	.370
Transient relief or recurrence, n/N (%)	1/8 (12.5)	2/15 (13.3)	Risk difference −0.8% (−27.4 to 35.1)	>.999
No meaningful improvement, n/N (%)	2/8 (25.0)	4/15 (26.7)	Risk difference −1.7% (−32.6 to 35.9)	>.999
Medication reduction, n/N (%)	5/8 (62.5)	11/15 (73.3)	Risk difference −10.8% (−46.4 to 23.9)	.657
Off pain medications, n/N (%)	3/8 (37.5)	2/15 (13.3)	Risk difference +24.2% (−10.0 to 57.5)	.297

Clinical follow-up is reported as median (IQR). Categorical variables are reported as n/N (%). Effect estimates are reported as risk differences for categorical variables and median difference for clinical follow-up, calculated as fusion minus nonfusion. Risk differences use Newcombe 95% CIs. The median difference for clinical follow-up is reported with a bootstrap 95% CI. *P* values are exploratory and were not adjusted for multiple comparisons. Any durable pain relief includes complete and partial durable responses. Transient relief or recurrence includes initial improvement followed by recurrence or loss of response. No meaningful improvement includes none, persistent pain, or failure. Medication reduction indicates any documented decrease in pain medication use and is reported separately from complete discontinuation.

### Radiographic Outcomes and Deformity Events

Radiographic outcomes are summarized in Table 4. Paired preoperative and postoperative standing radiographs were available in 7 of 8 fusion patients (87.5%) and 3 of 15 nonfusion patients (20.0%). Because standardized paired standing radiographs were limited in the historical nonfusion cohort, continuous alignment comparisons were performed using the paired X-ray subset, whereas deformity events were assessed using all evaluable nonfusion patients.

**TABLE 4.** Radiographic Outcomes and Deformity Events

Variable	Fusion cohort	Nonfusion comparator	Difference, fusion minus nonfusion (95% CI)	*P* value
Paired pre/post X-rays available, n/N (%)	7/8 (87.5)	3/15 (20.0)	Risk difference +67.5% (24.7 to 84.0)	**.006**
Paired X-ray measurements	n = 7	n = 3		
C2-C7 Cobb angle, mean ± SD	2.5 ± 23.1° → 18.3 ± 20.8°; Δ +15.7 ± 16.0°	−10.0 ± 7.9° → −18.7 ± 22.7°; Δ −8.7 ± 29.7°	Difference in Δ +24.4° (−40.3 to 89.1)	.383
CSVA, mean ± SD	33.5 ± 10.4 → 31.9 ± 11.1 mm; Δ −1.6 ± 8.0 mm	23.2 ± 4.8 → 58.2 ± 35.9 mm; Δ +34.9 ± 35.0 mm	Difference in Δ −36.5 mm (−120.9 to 47.9)	.067
T1 slope, mean ± SD	22.4 ± 12.3° → 28.6 ± 9.9°; Δ +6.2 ± 10.9°	14.0 ± 13.1° → 26.7 ± 16.3°; Δ +12.7 ± 12.0°	Difference in Δ −6.5° (−30.1 to 17.1)	.422
C2 slope, mean ± SD	19.9 ± 16.9° → 21.7 ± 11.7°; Δ +1.7 ± 8.3°	12.7 ± 7.5° → 45.0 ± 30.1°; Δ +32.3 ± 37.3°	Difference in Δ −30.6° (−120.7 to 59.5)	.117
TS-CL, mean ± SD	19.9 ± 11.9° → 10.4 ± 20.2°; Δ −9.5 ± 18.0°	24.0 ± 21.0° → 45.3 ± 26.8°; Δ +21.3 ± 38.4°	Difference in Δ −30.8° (−116.6 to 55.0)	.253
Postoperative threshold alignment				
Postoperative CSVA ≤40 mm, n/N (%)	8/8 (100)	1/3 (33.3)	Risk difference +66.7% (10.5 to 93.9)	.055
Postoperative TS-CL ≤20°, n/N (%)	7/8 (87.5)	0/3 (0)	Risk difference +87.5% (21.6 to 97.8)	**.024**
Preoperative kyphosis, n/N (%)	3/7 (42.9)	3/3 (100)	Risk difference −57.1% (−84.2 to 7.5)	—
Residual postoperative kyphosis despite improvement, n/N (%)	1/8 (12.5)	—	—	—
Radiographic deformity events	n = 8	All evaluable nonfusion patients		
New/progressive postoperative kyphosis or deformity, n/N (%)	0/8 (0)	5/12 (41.7)	Risk difference −41.7% (−68.0 to −2.3)	.055
Postoperative kyphosis documented in imaging/chart, n/N (%)	0/8 (0)	8/15 (53.3)	Risk difference −53.3% (−75.2 to −13.4)	**.019**
Reoperation for deformity/instability/radiographic failure, n/N (%)	1/8 (12.5)	5/15 (33.3)	Risk difference −20.8% (−47.8 to 18.2)	.369

CSVA, cervical sagittal vertical axis; TS-CL, T1 slope minus cervical lordosis.

Radiographic variables use a lordosis-positive convention; negative C2-C7 Cobb values indicate kyphosis. Paired X-ray measurements are shown as preoperative → postoperative values with mean change. Effect estimates are calculated as fusion minus nonfusion. For paired radiographic measurements, effect estimates represent the between-cohort difference in mean change and use Welch 95% CIs. For categorical outcomes, effect estimates are risk differences using Newcombe 95% CIs. *P* values are exploratory and were not adjusted for multiple comparisons. Fusion patient 1 had postoperative-only imaging and was excluded from paired-change calculations but included in postoperative threshold analyses. The nonfusion comparator includes the paired X-ray subset for continuous measurements and all evaluable nonfusion patients for deformity-event analyses. Residual postoperative kyphosis after fusion was not classified as new/progressive deformity if alignment improved from baseline. Bold denotes *P* value less than .05, indicating statistical significance.

In the fusion cohort, paired radiographic measurements demonstrated preservation or improvement of cervical sagittal alignment. Mean C2-C7 Cobb angle improved from 2.5° ± 23.1° preoperatively to 18.3° ± 20.8° postoperatively, corresponding to a mean increase in lordosis of 15.7° ± 16.0°. Mean CSVA remained stable, changing from 33.5 ± 10.4 mm to 31.9 ± 11.1 mm. Mean T1 slope increased from 22.4° ± 12.3° to 28.6° ± 9.9°, whereas C2 slope changed minimally from 19.9° ± 16.9° to 21.7° ± 11.7°. Mean TS-CL improved from 19.9° ± 11.9° to 10.4° ± 20.2°. At last radiographic follow-up, all fusion patients demonstrated arthrodesis.

Three fusion patients demonstrated preoperative kyphosis. All 3 improved postoperatively, although one had residual kyphotic alignment despite correction. Postoperatively, all fusion patients maintained CSVA ≤40 mm, and 7 of 8 patients (87.5%) had TS-CL ≤20°. No fusion patient developed new or progressive postoperative kyphosis or delayed deformity requiring corrective fusion.

In the paired nonfusion X-ray subset, mean C2-C7 alignment worsened from −10.0° ± 7.9° preoperatively to −18.7° ± 22.7° postoperatively, whereas CSVA increased from 23.2 ± 4.8 mm to 58.2 ± 35.9 mm. Only 1 of 3 patients (33.3%) maintained postoperative CSVA ≤40 mm, and no patient in this subset had postoperative TS-CL ≤20°.

**FIGURE 2.**
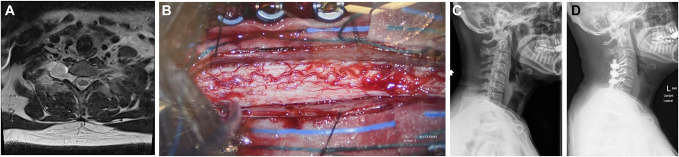
Representative case of DREZ lesioning with concurrent posterior instrumented fusion. **A**, Axial T2-weighted MRI at the T1 foramen demonstrating a left-sided pseudomeningocele consistent with T1 nerve root avulsion. **B**, Intraoperative photograph demonstrating dorsal root avulsion with bilateral posterior exposure and instrumentation. **C**, Preoperative lateral radiograph of a 44-year-old man with left brachial plexus avulsion after a bicycle accident 6 years before DREZ surgery, demonstrating kyphotic alignment. C2-C7 Cobb angle −12°, CSVA 31 mm, T1 slope 15°, C2 slope 4°. **D**, Postoperative lateral radiograph after C5-T1 DREZ lesioning with C4-T3 posterior instrumented fusion demonstrating restoration of cervical lordosis. C2-C7 Cobb angle 31°, CSVA 7.5 mm, T1 slope 16°, C2 slope 11°. CSVA, cervical sagittal vertical axis; DREZ, dorsal root entry zone.

Among all evaluable nonfusion patients, new or progressive postoperative kyphosis/deformity was documented in 5 of 12 patients (41.7%), and postoperative kyphosis was documented by imaging or chart review in 8 of 15 patients (53.3%). Reoperation for deformity, instability, or radiographic failure occurred in 5 of 15 patients (33.3%). Representative cases demonstrating progressive postinjury kyphosis and delayed deformity after nonfusion DREZ lesioning are shown in Figure 1. Fusion cohort alignment preservation and correction are shown in Figure 2.

## DISCUSSION

In this retrospective cohort of patients undergoing cervical or cervicothoracic DREZ lesioning for refractory deafferentation pain, concurrent posterior instrumented fusion was associated with preservation or improvement of cervical sagittal alignment despite more extensive posterior exposure. Fusion patients demonstrated increased C2-C7 lordosis, stable CSVA, and improved TS-CL. No patient in the fusion cohort developed new or progressive postoperative kyphosis or delayed deformity requiring corrective fusion. By contrast, the historical nonfusion cohort demonstrated higher rates of postoperative kyphosis, progressive deformity, and reoperation for deformity, instability, or radiographic failure.

### Cervical Deformity in Brachial Plexus Avulsion

A key finding of this study is that cervical kyphosis may precede index DREZ surgery. Preoperative kyphosis was present in 3 of 7 fusion patients and in all 3 nonfusion patients in the paired X-ray subset. In addition, representative cases demonstrated progressive kyphotic deformity during the chronic interval after injury. These observations support the hypothesis that traumatic brachial plexus avulsion may predispose to cervical malalignment before posterior surgical exposure. The mechanism is likely multifactorial. High-energy traction injuries may produce occult ligamentous strain, whereas chronic denervation, paraspinal muscle atrophy, shoulder girdle imbalance, and asymmetric loading may progressively alter cervical posture. Many DREZ patients present years after injury, often with flail or amputated extremities and long-standing compensatory mechanics.^15^ The cervical spine may therefore be biomechanically vulnerable before DREZ exposure. Multilevel laminectomy, particularly across the cervicothoracic junction, may further destabilize this vulnerable segment and accelerate deformity progression.

### Postlaminectomy Kyphosis and the Role of Fusion

Postlaminectomy kyphosis is a recognized complication after multilevel cervical posterior decompression.^16,17^ Although this risk is well described in other cervical pathologies, its relevance to DREZ lesioning has been less emphasized. DREZ surgery often requires multilevel dorsal cord exposure, and in brachial plexus avulsion the anatomy may be distorted by atretic roots, pseudomeningoceles, arachnoid scarring, cord rotation, or partial avulsion. Disruption of the posterior tension band predisposes to progressive sagittal malalignment, which may progress over years and result in pain, neurological compromise, and functional decline.^18^ Patients with nerve root avulsions demonstrate atrophy of the posterior paraspinal muscles, including multifidus and semispinalis cervicis, further reducing intrinsic cervical stability.^8^ This baseline loss of muscular support is compounded by the extensive dorsal exposure required for DREZ, which frequently spans multiple levels and crosses the cervicothoracic junction, a region subject to substantial biomechanical stress.

Sagittal alignment was largely preserved in the fusion cohort. The fusion cohort underwent full bilateral laminectomy exposure in all cases, and despite this more extensive exposure, postoperative alignment remained within accepted thresholds in most patients. All fusion patients maintained CSVA ≤40 mm, and 7 of 8 maintained TS-CL ≤20°. One patient had residual postoperative kyphosis, but this represented improvement from severe preoperative kyphosis rather than new or progressive deformity. These findings suggest that concurrent fusion may mitigate the kyphotic failure pattern observed after nonfusion DREZ.

### Comparison With Historical Nonfusion DREZ

The nonfusion cohort had limited standardized radiographic follow-up, but the available data demonstrated a clinically important deformity signal. In the paired X-ray subset, postoperative alignment worsened, with increased kyphosis, increased CSVA, and worsening TS-CL. Across the historical nonfusion cohort, postoperative kyphosis was documented in 8 of 15 patients (53.3%), and new or progressive postoperative kyphosis/deformity was documented in 5 of 12 evaluable patients (41.7%). Reoperation for deformity, instability, or radiographic failure occurred in 5 of 15 patients (33.3%). These findings should be interpreted cautiously because the nonfusion cohort was historical, heterogeneous, and incompletely imaged. Nevertheless, the pattern is clinically consistent: patients treated without fusion were more likely to demonstrate delayed kyphosis, instability, or deformity-related reoperation. This comparison supports the structural rationale for selective fusion when DREZ exposure is extensive or crosses the cervicothoracic junction.

### Pain Outcomes

Pain outcomes remained heterogeneous in both cohorts and were broadly consistent with previous DREZ experience.^19-21^ Durable pain relief occurred in approximately two-thirds of patients in both groups. Complete durable relief was numerically more frequent in the fusion cohort, but this study was not powered to determine whether fusion influenced analgesic efficacy.

The main observed benefit of fusion was structural rather than analgesic. Fusion allowed extensive exposure and lesioning while maintaining alignment and preventing delayed kyphotic progression. Patients should therefore be counseled that fusion may reduce deformity risk but does not eliminate the variable pain response inherent to DREZ lesioning.

### Surgical Exposure Considerations

Hemilaminectomy and laminoplasty may reduce posterior element disruption during DREZ lesioning, particularly for unilateral pathology, and laminoplasty may reduce postlaminectomy deformity risk in selected cervical procedures. However, their role in cervicothoracic DREZ lesioning is less straightforward. DREZ localization may be challenging after traumatic root avulsion because of atretic rootlets, arachnoid scarring, pseudomeningocele, cord rotation, or partial avulsion. In these cases, broader exposure may improve visualization of the dorsolateral sulcus and permit comparison of injured and intact rootlet anatomy. When exposure spans multiple levels or crosses the cervicothoracic junction, this benefit must be weighed against the known risk of postlaminectomy kyphosis, a concern even in elective degenerative cervical surgery and potentially greater in patients with traumatic denervation and abnormal cervicothoracic loading.^16^

Concurrent posterior fusion shifts this balance by allowing wide dorsal exposure while stabilizing the posterior tension band. In the present cohort, the motion-preservation advantage of laminoplasty was less relevant because posterior arthrodesis was performed as part of the index procedure. Fusion also introduces morbidity, including loss of motion, adjacent segment degeneration, and instrumentation-related complications.^22,23^ In this series, 1 patient required revision for instrumentation failure because of pedicle screw fracture. All fusion constructs were multilevel and crossed or stabilized the cervicothoracic junction, and greater fusion length has been associated with increased hospitalization and readmission after posterior cervical fusion.^24^ Therefore, concurrent fusion should not be viewed as routine for all DREZ cases, but as a selective strategy when extensive multilevel exposure, cervicothoracic junction involvement, preoperative kyphosis, or interval deformity progression increases the risk of postoperative structural failure.

### Limitations

This study is limited by its small sample size, single-center retrospective design, and historical nonfusion comparator. Radiographic follow-up was incomplete and heterogeneous in the nonfusion cohort, limiting quantitative alignment comparisons and requiring some deformity outcomes to be identified from imaging reports, outside documentation, or clinical notes. Follow-up duration was sufficient to detect early deformity but may underestimate late progression, adjacent-segment degeneration, or delayed instrumentation complications. Pain outcomes were abstracted retrospectively rather than using standardized prospective pain instruments. Larger multicenter studies with standardized radiographic follow-up are needed to refine indications for concurrent fusion during DREZ lesioning.

## CONCLUSION

Concurrent posterior instrumented fusion during cervicothoracic DREZ lesioning was associated with preservation of sagittal alignment and no cases of new or progressive postoperative kyphosis. Pain outcomes remained variable, suggesting that the primary benefit of fusion was structural rather than analgesic. Given the frequency of preexisting or delayed deformity in this population, concurrent fusion should be considered when DREZ exposure requires extensive multilevel decompression, particularly across the cervicothoracic junction.

## Ethics Approval

This study was approved by the Duke University Institutional Review Board name, Pro00090408.
